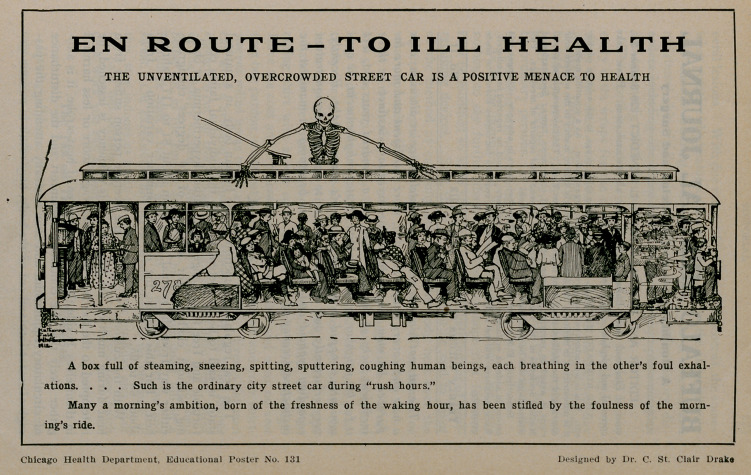# Description of a Simple Portable Apparatus for the Application of Plaster Jackets in Hyperextension

**Published:** 1913-06

**Authors:** Prescott Le Breton

**Affiliations:** Buffalo


### Description of a Simple Portable Apparatus for the Application of Plaster Jackets in Hyperextension.

BY PRESCOTT LE BRETON
Buffalo

MANY forms of apparatus have been described, the object of which was to allow the application of a jacket in the prone position, at the same time providing for a greater or less amount of hyperextension of the spine. The model, as shown in the illustrations, was devised by the writer, especially for cases of

Pott's disease in children, although it is useful in varied conditions. The principle is not new, but the mechanism differs, and its chief advantage is that it can be taken apart and put together in a few seconds and can be easily carried in one hand like an umbrella. The illustrations show it is effective in providing a jacket which gives fixation and hyperextension.

The lower part consists of two pieces of angle iron, each two feet long, bent at one end on the flat and fastened to a two-inch circular base of iron. One bar is screwed tight, the other loose, so that they may be separated any desired amount, to provide a firm base for the entire apparatus. The upright bar of iron is solid, five-eighths inch diameter and two feet long, and the lower end screws readily into a hole in the centre of the circular base. The upper end of the upright has an open slot for the crosspiece, and an offshoot with an enclosed slot for the reception of the end of the crosspiece. The crosspiece is a two-foot angle iron, one end of which rests in the open slot, extending beyond to be caught at the extreme end in the enclosed slot. The slots are wide, to allow one to place this upper bar readily in position. The illustrations show two indentations which prevent the ends of the bandage from slipping. When disconnected, the three parts, being straight and of the same length, are easily carried by hand.

The patient puts on a stockinette shirt as usual, and lies down as in the photograph, buttocks and shoulders resting on supports of some kind, to raise the back from the table. The apparatus is placed crosswise, the upper bar over the kyphos. A thick felt pad is held against the kyphos, and a broad muslin bandage placed against the felt, the ends coming up to the upper bar. The ends are tied with enough tension to bow the back to the desired degree. Sheet wadding, felt and plaster bandages are used as in any case. Later the bandage is cut where it emerges and the holes covered by a layer of plaster bandage.

It is evident that close application of the jacket is possible at the three essential points, *i. e.,* the kyphos posteriorly and the sternum and anterior spinous processes anteriorly. For high Pott's, where a jacket is continued above the shoulders or for acute disease in the cervical region, attended by pain, spasm, or abscess formation, the same apparatus may be used, modifying its position and possibly using two bandages for support, the edges of which could be stitched to the stockinette to hold them in the desired position. With an assistant at the head and another at the feet, traction may be provided as the jacket is applied. The upright position by Sayre's suspension is often inconvenient, owing to the age of the patient or his condition, hence the advantage of an appliance providing for the application of jackets in the prone position, preferably on the back and not on the face.

---

General Paralysis in Dogs. L. Marchand and G. Petit, *Arch. Internat. de Neurol.,* November, 1912, recognize clinically, a commoner dementia type and an epileptic. Pathologically, there is diffuse, sub-acute meningitis and encephalitis, and often there is a cerebellar, bulbar or cord lesion. The symptoms are described quite in human terms, as loss of memory, filthy habits, boulimia, etc. These observations are held to indicate the importance of vaguely understood, non-syphilitic causes in producing general paralysis. However, in the light of recent discoveries, syphilis cannot be regarded as an exclusively human disease, and further research is necessary to show how its manifestations, as well as those of other diseases, are modified by a difference of genus in the hosts.

---

Hyperthyroidism Precipitated by Iodine. Jervell, *Forhand. i det. Med. Srlskab,* 1912, page 256. Gastro-enterostomy was done for obstructive gastric cancer, the skin being disinfected with iodine. The patient was a woman aged 64, with a small elastic left-sided goitre which had never given symptoms. On the third day the pulse gradually rose to 180 and reached 200 on the fourth. Other but not all signs of Graves's disease were present. Death occurred on the nineteenth day, although digalen had temporarily brought the pulse down. The heart was slightly dilated, flaccid, and there was a small embolus in the right pulmonary artery. Iodine was absent in the urine from the 7th to the 10th day, present from the 11th to the 15th.

---

Cretinism Relieved by Thyroid Extract. F. Lee Stone of Beaver Crossing, Neb., reports the case of a child eight months old, born of German parents. *Western Med. Rev.,* April, 1913.

## Figures and Tables

**Figure f1:**
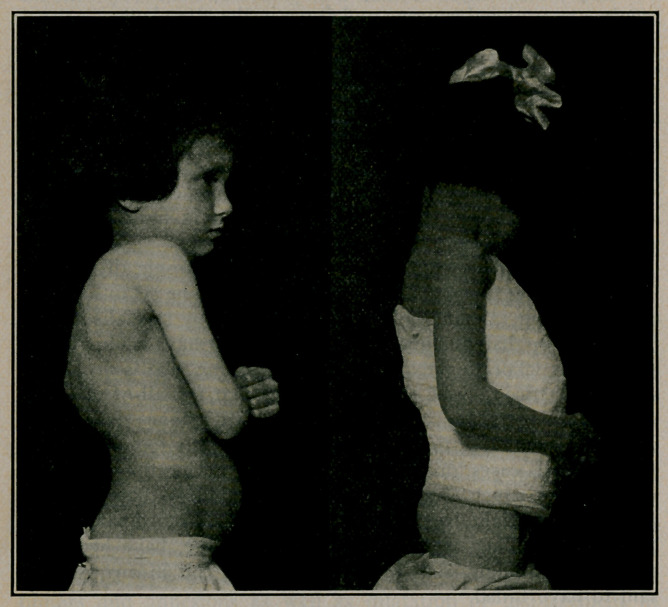


**Figure f2:**
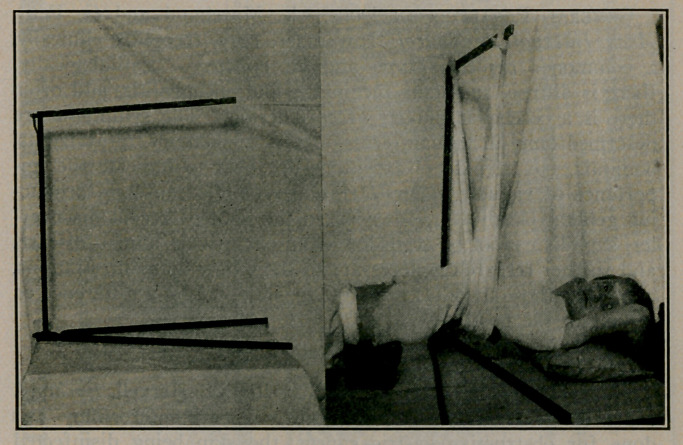


**Figure f3:**